# Editorial Expression of Concern: Ets-1 mediates upregulation of Mcl-1 downstream of XBP-1 in human melanoma cells upon ER stress

**DOI:** 10.1038/s41388-025-03436-7

**Published:** 2025-05-08

**Authors:** L. Dong, C. C. Jiang, R. F. Thorne, A. Croft, F. Yang, H. Liu, C. E. de Bock, P. Hersey, X. D. Zhang

**Affiliations:** 1 Immunology and Oncology Unit, Calvary Mater Newcastle Hospital, Newcastle, New South Wales Australia; 2 Cancer Research Unit, School of Biomedical Sciences, University of Newcastle, Newcastle, New South Wales Australia

Editorial Expression of Concern to: *Oncogene* 10.1038/onc.2011.87, published online 21 March 2011

The Editors-in-Chief would like to alert the readers that concerns have been raised regarding some of the data presented in this article. Speficically:

- Fig. 2c Mel-CV and MM200 images appear highly similar;
- Fig. 5a IRE1α MM200 (lanes 4-6) and 6e Ets-1 Mel-KD blots appear highly similar.

The authors have been able to replicate the data, but due to the age of the article, the original data could not be recovered. Readers are therefore advised to interpret these results with caution.

C. E. de Bock agrees to this Editorial Expression of Concern. R. F. Thorne does not agree to this Editorial Expression of Concern. The other authors have not responded to any correspondence from the editor or publisher about this Editorial Expression of Concern.

